# Research on Configuration Constraints of Airborne Bistatic SARs

**DOI:** 10.3390/s22176534

**Published:** 2022-08-30

**Authors:** Yidi Chen, Renwen Chen, Hao Liu, Jiapeng Guo, Yujie Wang, Junyi Zhang

**Affiliations:** College of Aerospace Engineering, Nanjing University of Aeronautics and Astronautics, Nanjing 210016, China

**Keywords:** bistatic SAR, airborne, spatial configuration, radar imaging

## Abstract

Based on the analysis of the airborne bistatic synthetic aperture radar (SAR) imaging geometric mode, an extended nonlinear chirp scaling algorithm is employed to simulate and verify the imaging effect of the bistatic SARs. A gradient theory-based two-dimensional resolution bistatic SAR model is proposed, and the constraints of the multi-platform flight trajectory parameters meeting the imaging accuracy of the bistatic SAR are analyzed. Finally, through the bistatic SAR imaging simulation of cooperative flight trajectories under various situations, the spatial configuration constraint envelope between the flight vehicles to achieve the optimal resolution is revealed. The results of this paper will provide a theoretical reference for the SAR application in formation flight control.

## 1. Introduction

Bistatic synthetic aperture radar (SAR) puts the receiver and transmitter on different platforms. The transmitter actively transmits radar signals to the target area and the receiver passively receives the echo signals to achieve high-resolution two-dimensional imaging of the target area [1,2]. Bistatic SAR has outstanding advantages such as good concealment, strong target detection ability, strong anti-interference ability, forward-looking imaging [3], etc., and thus has a good application prospect.

Many researchers have made great efforts to promote bistatic SAR imaging accuracy and range. Martino studied an imaging geometry model and the processing algorithm of a spaceborne bistatic SAR system for formation flight in large-baseline mode [4], and Servidia proposed a control rate method of configuration reconstruction of a spaceborne bistatic SAR system for formation flight in different Earth observation modes [5]. Lu established an imaging model of the GEO-LEO bistatic interferometric SAR system, designed its formation configuration, and analyzed its interferometric measurement accuracy [6]. The above methods are all based on the satellite platform with an accurate and controllable trajectory. However, it will not be suitable in some cases when the flight trajectory is unknown in advance. To overcome this problem, Li established an imaging geometric model of a missile-borne forward bistatic SAR system and analyzed the influence relationship between the two-dimensional resolution and system parameters [7]. Meng established a general bistatic forward SAR signal model and analyzed the azimuth space variation effect and its spectral characteristics [8]. Ding analyzed a bistatic SAR signal model in the special mode, when the transmitting platform horizontally flies while the receiving platform pitches down, and proposed an extended chirp scaling (CS) imaging algorithm [9]. S. Li analyzed a signal model under the non-parallel flight trajectory of the receiving platform, and a nonlinear CS (NLCS) imaging algorithm based on a frequency extension was proposed [10].

The high mobility of the moving platform makes the bistatic SAR imaging resolution change significantly. Therefore, it is more important than ever to obtain the balance between the high mobility of the moving platform and the optimal resolution of bistatic SAR imaging by reasonably planning the motion trajectory of the transceiver platform. However, because the resolution of the bistatic SAR forward-looking imaging is significantly influenced by the geometric model parameters of the platform and the target, for practical applications, it is necessary to analyze the influence of the parameters of the receiving platform and the target geometric model and find the configuration boundary of the moving platform under the constraints of the optimal or necessary resolution.

This paper investigates the airborne bistatic SAR imaging geometry model and the extended NLCS algorithm and verifies the imaging effects by simulation. Under the premise of the imaging effect, the constraint relationship between the aircraft spatial configuration and the imaging resolution is analyzed. Finally, the configuration parameter envelope that meets with the predefined resolution is obtained by a bistatic SAR imaging simulation through all the cooperative flight routes.

## 2. Imaging Geometry Model of Bistatic SAR

**Figure 1:**
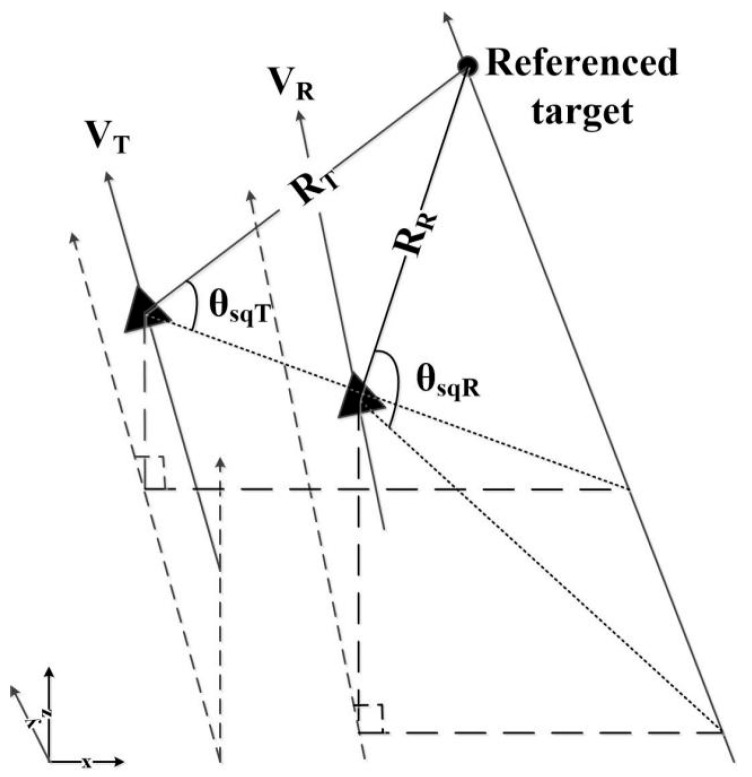
Imaging geometry model of bistatic forward-looking SAR.

A general bistatic geometric model [11] is shown in Figure 1. It is assumed that the transmitter and the receiver fly along an unparallel straight line at a constant speed. The transmitter’s flight speed is *V_T_* and the receiver’s is *V_R_*, and the two speeds are different. A right-hand coordinate system is adopted, and the x-y plane is the ground plane, at which the target point is located. The flight direction of the transmitter is parallel to the *y* axis.

In the bistatic model, both the radar wave beams of the transmitter and the receiver are at the same direction and cover the reference targets. The instantaneous slope range between the transmitter and the target is $R_{T}\left(\eta \right)$, the corresponding receiving instantaneous slope range is $R_{R}\left(\eta \right)$, the time at the range direction is τ, and the time at azimuth direction is η. When the radar beam center irradiates on the reference target point, the azimuth time is η=0, at which the transmitting angle and receiving angle are $\theta_{sqT}$ and $\theta_{sqR}$, respectively.

Assuming that the transmitted broadband signal is s(τ), up-conversion of the signal at frequency $f_{o}$ will result in

(1) st(τ)=Re{s(τ)exp(j2πf0τ)}

and the two-way range R(η) of the echo signal from the target will be

(2) $$s_{r}\left(\tau ,\eta \right)=w_{az}s_{t}\left(\tau -\frac{R\left(\eta \right)}{c}\right)$$

where $w_{az}$ is the azimuth envelope, and c is the speed of light. Here, the amplitude of the signal is ignored. R(η) [12] may be described as

(3) $$\begin{array}{ll} R\left(\eta \right) & =R_{T}\left(\eta \right)+R_{R}\left(\eta \right)\\ & =\sqrt{V_{T}^{2}\eta^{2}+R_{Tcen}^{2}-2V_{T}\eta R_{Tcen}\sin\left(\theta_{sqT}\right)}\\ & \;+\sqrt{V_{R}^{2}\eta^{2}+R_{Rcen}^{2}-2V_{R}\eta R_{Rcen}\sin\left(\theta_{sqR}\right)} \end{array}$$

In Equation (3), $R_{Tcen}$ is the range between the transmitting platform and the target at η=0 moment, and $R_{Rcen}$ is the range between the receiving platform and the target at η=0 moment.

Through down conversion, one can obtain the equation below

(4) $$s\left(\tau ,\eta \right)=w_{az}\left(\eta \right)w_{r}\left(\tau -\frac{R\left(\eta \right)}{c}\right)\exp\left\{-j2\pi \frac{R\left(\eta \right)}{\lambda }\right\}$$

where $w_{r}\left(*\right)$ is the range envelope and $w_{az}\left(*\right)$ is the azimuth envelope, which are determined by the composed antenna azimuth diagram.

## 3. Imaging Principle Model of Bistatic SAR

### 3.1. Analysis of Bistatic SAR Echo Signal

When the bistatic SAR echo signal model is analyzed, the time domain expression of target signal may be written as [11]

(5) $$s_{A}\left(\tau ,\eta \right)=w_{az}\left(\eta \right)s_{r}\left(\tau -\frac{R\left(\eta \right)}{c}\right)\exp\left\{-j2\pi \frac{R_{1}\left(\eta \right)}{\lambda }\right\}$$

where

(6) $$R_{1}\left(\eta \right)=R_{cen}+k_{2}\eta^{2}+k_{3}\eta^{3}+k_{4}\eta^{4}+\ldots$$

$R_{1}\left(\eta \right)$ is the range after the linear components’ compensation, and Fourier series expansion is performed at azimuth zero, $R_{cen}$ is the sum of $R_{Tcen}$ and $R_{Rcen}$.

Using series inversion method [11], the two-dimensional spectrum expression of echo signal can be derived as [11]

(7) $$S\left(f_{\tau },f_{\eta }\right)=W_{r}\left(f_{\tau }\right)W_{az}\left(f_{\eta }+\left(f_{o}+f_{\tau }\right)\frac{k_{1}}{c}\right)\exp\left\{j\phi \left(f_{\tau },f_{\eta }\right)\right\}$$

where $\phi \left(f_{\tau },f_{\eta }\right)$ is the phase [11]

(8) $$\phi \left(f_{\tau },f_{\eta }\right)=-2\pi \left(\frac{f_{o}+f_{\tau }}{c}\right)R_{cen}-\frac{\pi f_{\tau }^{2}}{K_{r}}\;\;\;\;\;\;\;\;\;\;\;\;\;+2\pi \frac{c}{4k_{2}\left(f_{o}+f_{\tau }\right)}{\left(f_{\eta }+\left(f_{o}+f_{\tau }\right)\frac{k_{1}}{c}\right)}^{2}\;\;\;\;\;\;\;\;\;\;\;\;\;+2\pi \frac{c^{2}k_{3}}{8k_{2}^{3}{\left(f_{o}+f_{\tau }\right)}^{2}}{\left(f_{\eta }+\left(f_{o}+f_{\tau }\right)\frac{k_{1}}{c}\right)}^{3}\;\;\;\;\;\;\;\;\;\;\;\;\;\;\;+2\pi \frac{c^{3}\left(9k_{3}^{2}-4k_{2}k_{4}\right)}{64k_{2}^{5}{\left(f_{o}+f_{\tau }\right)}^{3}}{\left(f_{\eta }+\left(f_{o}+f_{\tau }\right)\frac{k_{1}}{c}\right)}^{4}\;$$

### 3.2. Bistatic SAR Imaging Algorithm

For the bistatic SAR imaging processing algorithm, Wang et al. derived an accurate bistatic point target reference spectrum and presented a frequency domain-based focusing algorithm [13]; some algorithms such as fast filtering back-projection and a nonlinear CS (NLCS) algorithm were proposed in papers [14,15,16,17,18,19,20,21] in which the bistatic SAR imaging of aircraft platform is processed based on the extended NLCS algorithm.

**Figure 2:**
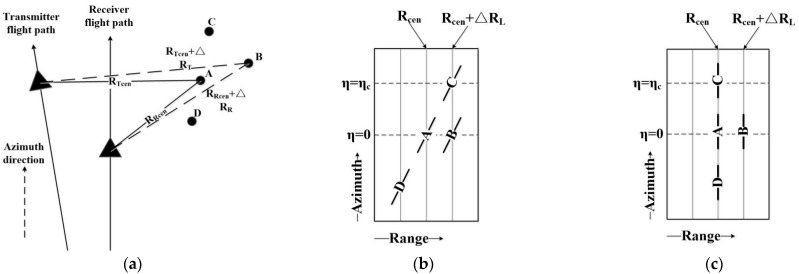
Description of RCMC. (**a**) Bistatic imaging geometry model, (**b**) before LRCMC, (**c**) after LRCMC.

Suppose that four point targets are placed in the bistatic SAR imaging model. When the compound beam center passes through target B and target C, the minimum slope ranges of the two targets are the same, and the compound beam center passes through target B and target A at the same time. The slope of linear range cell migration (LRCM) of targets A, C, and D is consistent, as shown in Figure 2.

The signal expression of target point A after linear range cell migration correction (LRCMC) and linear phase removal is as follows [12]

(9) $$s_{A}\left(\tau ,\eta \right)\approx \rho_{r}\left\{\tau -\frac{R_{lrcmA}\left(\eta \right)}{C}\right\}w_{az}\left(\eta \right)\exp\left\{-j2\pi \frac{R_{lrcmA}\left(\eta \right)}{\lambda }\right\}$$

where $\rho_{r}\left(*\right)$ is the range pulse compression envelope.

Similarly, the signal expression of target point C after LRCMC may be deduced as follows

(10) $$s_{c}\left(\tau ,\eta \right)\approx \rho_{r}\left\{\tau -\frac{R_{lrcmC}\left(\eta \right)}{c}\right\}w_{az}\left(\eta -\eta_{C}\right)\exp\left\{-j2\pi \frac{R_{lrcmC}\left(\eta \right)}{\lambda }\right\}$$

where

(11) $$R_{lrcmC}\left(\eta \right)=R_{cenA}+k_{C2}{\left(\eta -\eta_{C}\right)}^{2}+k_{C3}{\left(\eta -\eta_{C}\right)}^{3}+k_{C4}{\left(\eta -\eta_{C}\right)}^{4}+\ldots$$

Because $k_{A2}\neq k_{C2}$, the Doppler frequency modulation slope of target point A and target point C is inconsistent, so a single azimuth matched filter cannot be applied for azimuth compression. Before azimuth matched filtering, the quadratic compression term of range and azimuth coupling is removed.

NLCS algorithm’s disturbance function is used to equalize the quadratic phase term of the target point, and the disturbance function of target point A is introduced as follows [12]

(12) $$s_{Apert}\left(\tau ,\eta \right)=s_{A}\left(\tau ,\eta \right)\exp\left\{j\pi \alpha \eta^{3}\right\}$$

where

(13) $$\alpha =\frac{1}{3}\left[\frac{V_{T}^{2}\cos^{2}\theta_{sqT}}{\lambda R_{TcenA}R_{cenA}}+\frac{V_{R}^{2}\cos^{2}\theta_{sqR}}{\lambda R_{RcenA}R_{cenA}}\right]\times \left(V_{T}\sin\theta_{sqT}+V_{R}\sin\theta_{sqR}\right)$$

Then, after the phase term of target point C is multiplied by the disturbance function

(14) $$s_{Cpert}^{\prime }\left(\tau ,\eta_{1}\right)\approx \rho_{r}\left(\tau -\frac{R_{cenA}}{c}\right)w_{az}\left(\eta_{1}\right)\;\;\;\;\;\;\;\;\;\;\;\;\;\;\;\times \exp\left\{j\pi \alpha \eta^{3}\right\}\exp\left\{j\pi \alpha 3\eta_{C}^{2}\eta_{1}\right\}\exp\left\{j\pi \alpha \eta_{C}^{3}\right\}\;\;\;\;\;\;\;\;\;\;\;\;\;\;\;\times \exp\left\{j\pi \left(\frac{V_{T}^{2}\cos^{2}\theta_{sqT}}{R_{TcenA}}+\frac{V_{R}^{2}\cos^{2}\theta_{sqR}}{R_{RcenA}}\right)\eta_{1}^{2}\right\}$$

Among them, the first term is the cubic phase modulation term, and all target points are consistent.

The second term is the Doppler offset, and all target points have the same offset. Usually, the azimuth sampling frequency is 20% greater than the Doppler bandwidth, which will not cause aliasing [22]. Therefore, it is necessary to control the offset within a certain range to avoid aliasing.

The third term is a constant, which depends on the position of the target point and has no effect on the focus of the other point.

The fourth term is azimuth linear frequency modulation, and all targets are tuned at the same frequency.

Because the disturbance function only considers the quadratic phase term and does not consider the higher-order term, the higher-order phase higher than the quadratic is not equalized. Therefore, the influence of the higher-order term should be considered in azimuth matched filtering. Meanwhile, in the shift-invariant region of imaging, the influence of higher-order terms can be ignored. Therefore, only the target point A can be used to deduce the azimuth matched filter function.

The azimuth Fourier transform of the target point A is carried out as [12]

(15) $$S_{azA}\left(\tau ,f_{\eta }\right)\approx \int w_{r}\left(\tau -\frac{R_{cenA}}{c}\right)w_{az}\left(\eta \right)\exp\left\{-j2\pi f_{\eta }\eta \right\}\;\;\;\;\;\;\;\;\;\;\;\;\;\;\times \exp\left\{-j\frac{2\pi }{\lambda }\left(k_{A2}\eta^{2}+k_{A3}\eta^{3}+k_{A4}\eta^{4}\right)+j\pi \alpha \eta^{3}\right\}d\eta$$

Using the stationary phase principle and series inversion, the azimuth matched filter is obtained as the conjugate term, as is shown below

(16) $$h_{amf}\left(f_{\eta }\right)=\exp\left\{-j\phi_{amf}\left(\eta \left(f_{\eta }\right)\right)\right\}$$

where

(17) $$\phi_{amf}\left(f_{\eta }\right)=-\frac{2\pi }{\lambda }\left(k_{A2}\eta^{2}+k_{A3}\eta^{3}+k_{A4}\eta^{4}\right)+\pi \alpha \eta^{3}-2\pi f_{\eta }\eta$$

and the relationship with frequency is shown in the following formula

(18) $$\eta =A_{1}f_{\eta }+A_{2}f_{\eta }^{2}+A_{3}f_{\eta }^{3}+\ldots$$

(19) $$a_{1}=-\frac{2}{\lambda }k_{A2}\;a_{2}=\left(\frac{3}{2}\alpha -\frac{3}{\lambda }k_{A3}\right)\;a_{3}=-\frac{4}{\lambda }k_{A4}$$

(20) $$A_{1}=\frac{1}{a_{1}}\;A_{2}=-\frac{a_{2}}{a_{1}^{3}}\;A_{3}=\frac{2a_{2}^{2}-a_{1}a_{3}}{a_{1}^{5}}$$

The azimuth matched filter under each range element can be calculated numerically (*k* value depends on the range element). Finally, the focused images of all range units of the whole shift-invariant scene are obtained.

**Figure 3:**
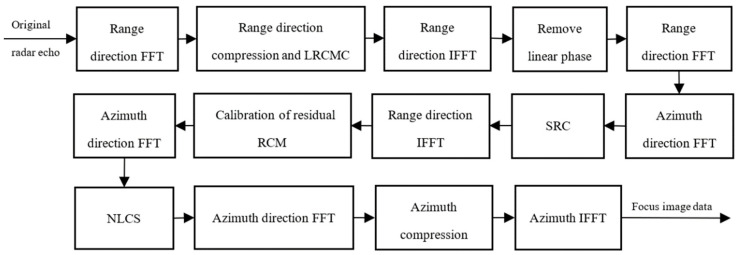
Flowchart of bistatic SAR imaging algorithm.

The flowchart of the proposed bistatic SAR imaging algorithm is shown in Figure 3. In the distance direction, the fast Fourier transform (FFT) algorithm is firstly performed on the original radar echo signal, and the echo signal is transformed into the frequency domain for processing. The received pulse is then compressed in the distance direction, so that the main energy of the signal is concentrated into a narrower duration. In order to eliminate the coupling between range and azimuth direction, the trajectory of the range migration curve is corrected to a straight line parallel to the azimuth, and LRCMC is used for correction. Then, perform inverse fast Fourier transform (IFFT) transformation on it, remove the linear phase, then FFT transformation. In the azimuth direction, first perform FFT transformation on the signal of the above operation and transform it into the frequency domain for processing. In order to eliminate the phase coupling distortion under strabismus or large aperture, the Second Range Compression (SRC) algorithm is used. The signal is then transformed into the time domain for residual calibration. In order to solve the nonlinear spatial variation of range migration (RCM) with range and the linear spatial variation of SRC with range of motion-invariant bistatic forward-looking SAR, the NLCS imaging algorithm is adopted. Then, perform FFT, azimuth compression, and IFFT transformation on it, and finally obtain focused image data.

### 3.3. Simulation Verification of Bistatic SAR Imaging Algorithm

**Figure 4:**
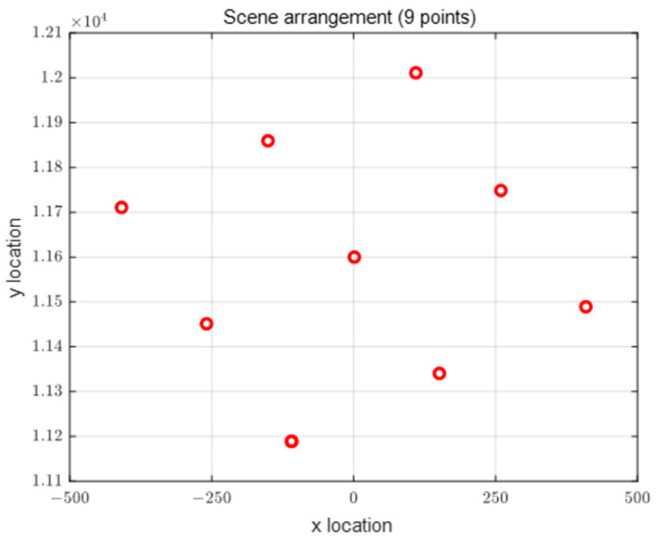
Layout of scenic spots.

The transmitter position error, receiver position error, and transmission line length measurement error affected the quality of imaging only slightly. All of these measurement errors caused an image shift along the range direction [23]. Therefore, the error analysis of bistatic SAR imaging is not discussed in the simulation. The configuration and system parameters are initialized as Table 1. The transverse range between the transmitting and the receiving platforms is 5 km, the transmitting platform is 1 km behind the receiving platform in longitude direction, and the heights of the two platforms are the same. Based on this configuration, the bistatic SAR imaging simulation verification is carried out. On the ground, nine points are arranged according to the receiver coordinate system, as shown in Figure 4.

**Figure 5:**
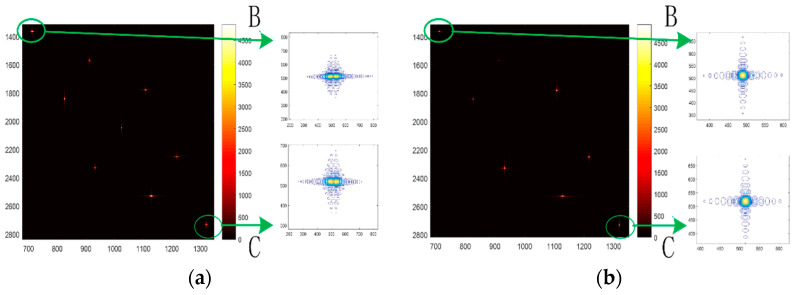
Simulation of scenic spots. (**a**)Traditional azimuth pulse compression, (**b**) pulse compression after space variation correction.

Points B and C are the edge points of the field-focusing diagram, as is shown in Figure 5. Figure 5a is the imaging effect diagram without the azimuth variation correction, and Figure 5b is the imaging effect diagram with the two-dimensional variation correction. As can be seen from the focusing effect, the azimuth focusing effect is good after the azimuth space variation correction, while the edge points without the two-dimensional space variation correction show the defocus phenomenon.

**Figure 6:**
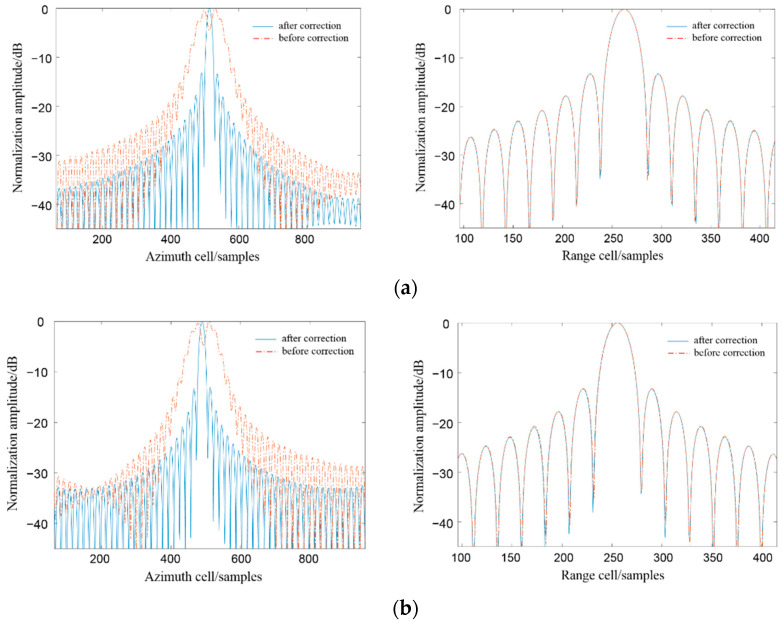
Schematic diagram of azimuth and range pulse at point B. (**a**) Schematic diagram of azimuth and range pulse at point C. (**b**) Schematic diagram of azimuth and range pulse at point B.

Figure 6 shows the pulse profiles of points B and C along the azimuth and range directions. The blue solid line and the orange dotted line represent the result after and before the correction, respectively. As can be seen from the figure, the two-dimensional focusing effect of the target point is better after the space variation correction, but without the space variation correction, the azimuth is raised to the first sidelobe, the peak sidelobe ratio is lost, and the azimuth resolution is decreased. In addition, it can be seen from the PSLR and ISLR calculation results in Table 2 that the focusing effect of the edge points of the dual or multiple base SAR imaging algorithm in this configuration is consistent with that of the scene center point, which fully demonstrates the feasibility of this configuration.

In addition, the peak sidelobe ratio and integral sidelobe ratio index parameters of point A, point B, and point C of the scenic spots in the central field were calculated, as shown in the following table. It can be seen that the focusing effect of points A, B, and C is good.

## 4. Analysis of Cooperative Configuration Constraints for Aircrafts

### 4.1. Analysis of Bistatic SAR Imaging Architecture

**Figure 7:**
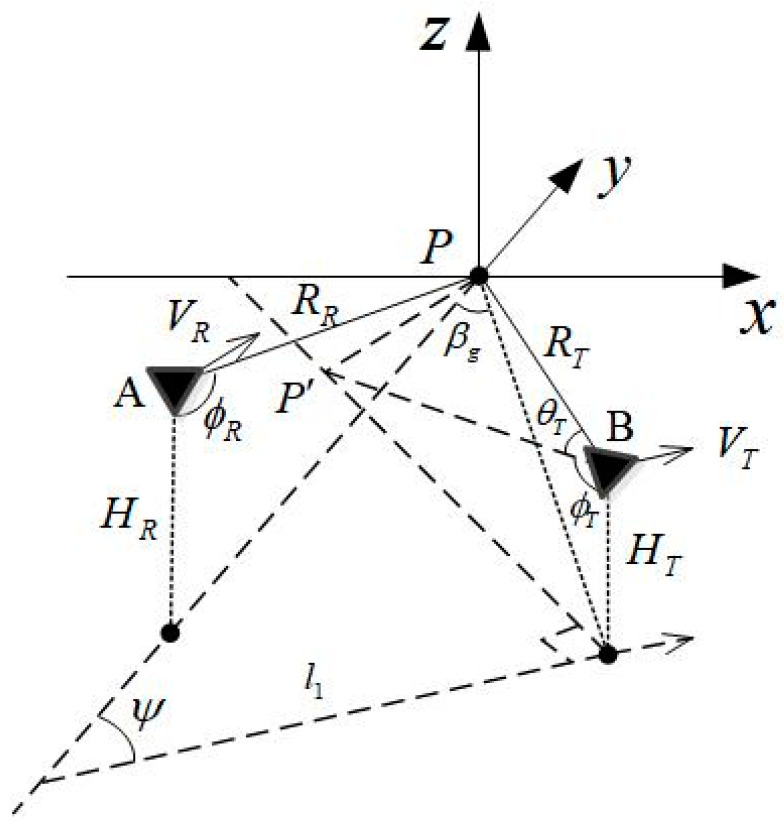
Geometric model of bistatic forward-looking SAR imaging.

The bistatic SAR imaging effect is mutually restricted with the spatial configuration of aircrafts, so it is necessary to select appropriate matching parameters between the imaging quality and the platform flight control to ensure a good configuration of the navigation control and to create conditions for high-precision imaging. Therefore, it is necessary to study the oblique angle of view, flight track, irradiation band width, and beam angle planning of the signals transmitted by the aircraft platform in cooperative flight to the target, as well as the accurate alignment and reception of the reflected signals of the target. The geometric model of the bistatic SAR forward-looking imaging of aircraft is shown in Figure 7.

Based on the simulation analysis of the imaging characteristics under dynamic conditions, the design method of the cooperative flight trajectory is proposed as follows:

1. First, determine the standard flight track of the radar signal receiving platform according to the flight destination.
2. According to the preset flight trajectory of the receiving platform, design the flight airspace range of the transmitting platform that meets the requirements of the imaging resolution.
3. Further, determine the actual flight envelope of the transmitting platform based on the flight airspace range of the transmitting platform in (2), considering the deviation of the actual navigation and control system of the transmitting platform, as well as the engineering constraints, such as the change of the range between the flight platform and the target, and the communication between the platforms.
4. Finally, plan the cooperative flight scheme and flight parameters of the receiving platform and the transmitting platform.

### 4.2. Resolution Analysis of Multi-Base SAR Imaging Based on Gradient Principle

Due to the influence of spatial relationship among target, transmitting platform, and receiving platform, the time resolution of aircraft bistatic SAR is firstly analyzed under given flight trajectory conditions in order to achieve two-dimensional high-resolution imaging.

According to the gradient principle, the forward-looking range resolution of the double base can be expressed as [24]

(21) $$\rho_{rg}=\frac{c}{B\left(i_{Rg}+i_{Tg}\right)}$$

$i_{R_{g}}$ and $i_{T_{g}}$, respectively, represent the ground projection of the range gradient of the transceiver platform, c represents the propagation speed of radar wave, B the signal bandwidth. $\rho_{rg}$ the direction of range resolution, and its modulus $\left|\rho_{rg}\right|$ is the size of range resolution, which reflects the ability of this configuration to distinguish targets in the horizontal plane. Then, on the premise of a certain signal bandwidth, $\left|\rho_{rg}\right|$ is related to the bistatic projection angle $\beta_{g}$ formed by the receiving platform and the target, as well as the projection component of the range gradient on the ground. All these factors are implicit and cannot be directly applied to the subsequent configuration design. Therefore, we convert the above formula to

(22) $$\left|\rho_{rg}\right|=\frac{c}{B\sqrt{\sin^{2}\varphi_{r}+\sin^{2}\varphi_{t}+2\sin\varphi_{r}\sin\varphi_{t}\cos\beta_{g}}}$$

According to the above formula, under the condition of a single variable, the larger $\beta_{g}$ is, the worse the ground range resolution will be. The smaller $\varphi_{t}$ is, or the higher the flight height $H_{t}$ of the aircraft is, the worse the ground range resolution will be.

According to the gradient principle, at the azimuth moment $t_{n}$, the azimuth resolution of the scene center point is [24]

(23) $$\rho_{ag}=1/(T_{a}\nabla f_{g})$$

where

(24) $$\nabla f_{g}=\frac{1}{\lambda }\left\{\frac{1}{R_{t}}\left[V_{tg}-\left(V_{tg}\cdot i_{Tg}\right)i_{Tg}\right]+\frac{1}{R_{r}}\left[V_{rg}-\left(V_{rg}\cdot i_{Rg}\right)i_{Rg}\right]\right\}$$

$\nabla f_{g}$ is the project of the Doppler gradient vector of the system on the ground. $V_{rg}$ and $V_{tg}$ represent the projection of the velocity vector of the receiver and the transmitter platform on the ground, respectively. $R_{r}$ and $R_{t}$ are the ranges between the transceiver platform and the target at the center of aperture, respectively. $T_{a}$ the synthetic aperture time. The direction of $\rho_{ag}$ is the direction of azimuth resolution, and its modulus $\left|\rho_{ag}\right|$ is the magnitude of azimuth resolution.

According to Equation (24), $\nabla f_{g}$ is composed of the system wavelength λ and the angular velocity vector of the transceiver platform, which is not only related to their modulus values but also related to their respective directions. For the convenience of analysis, $\nabla f_{g}$ can be updated as

(25) $$\nabla f_{g}=\nabla f_{tg}+\nabla f_{rg}=\frac{1}{\lambda R_{t}}(V_{tg}\cos\theta_{tg})i_{Twg}+\frac{1}{\lambda R_{r}}(V_{rg}\cos\varphi_{rg})i_{Rwg}$$

where $i_{Rwg}$ and $i_{Twg}$ is the projection on the ground of the angular velocity unit vector of the transceiver platform, respectively. $V_{tg}\cos\theta_{tg}∕\lambda R_{t}$ represents the Doppler component mode $\nabla f_{tg}$ provided by the transmitter, and $V_{rg}\cos\varphi_{rg}∕\lambda R_{r}$ represents the Doppler component mode $\nabla f_{rg}$ provided by the receiver. When the magnitude difference of the Doppler components provided by the transceiver platform is large, the azimuth resolution of the system can be approximately considered to be determined by the Doppler components of a single platform.

**Figure 8:**
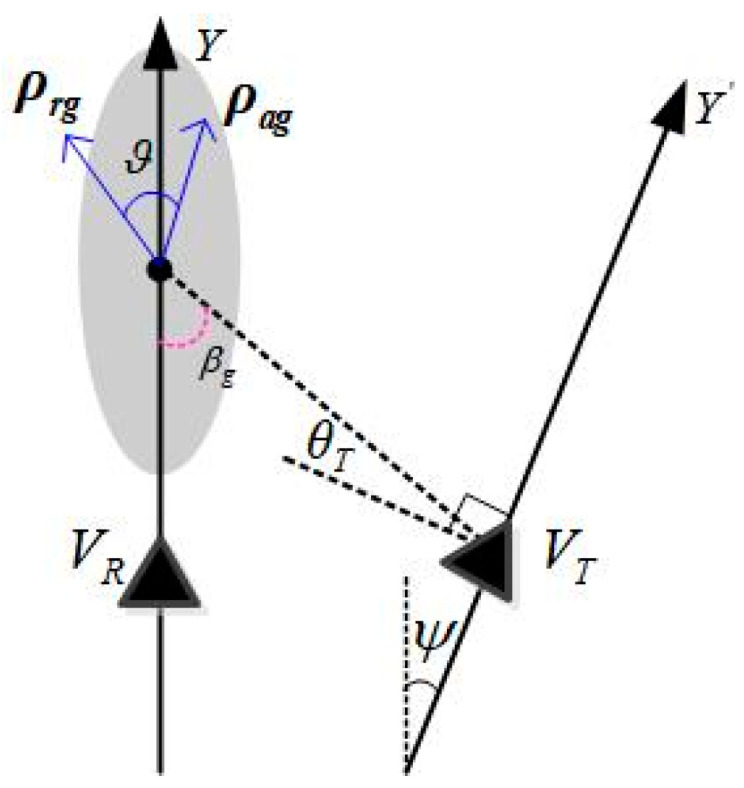
Schematic diagram of area of resolution element.

In order to further constrain the double-base resolution, a concept of resolving unit area is proposed, as shown in Figure 8. Because when the angle between range resolution direction and azimuth resolution direction is too small or too large, two-dimensional high-resolution images cannot be formed, the resolution unit area S can be defined by

(26) $$S=\frac{\left|\rho_{rg}\right|\cdot \left|\rho_{ag}\right|}{\sin\vartheta }$$

where ϑ is the included angle of range and azimuth resolution, that is, the included angle of direction $\rho_{rg}$ and $\rho_{ag}$. It can be found that when the range and azimuth resolutions are orthogonal, the two-dimensional resolution formed by the same resolution becomes the best.

In the bistatic forward configuration, the direction of the two-dimensional resolution cannot remain orthogonal all the time. Therefore, α needs to be rationally planned. When the target is far away, the included angle α of the 2D resolution of all points in the scene is evenly distributed, which can be designed according to the center point of the scene. However, when the target distance is relatively close, the distribution of α is not uniform due to the large Doppler gradient vector direction span of the receiver. In order to improve this situation, it is necessary to increase the Doppler component of the transmitter as much as possible by reducing the angle of view $\theta_{t}$ of the transmitter when the target is close to the target.

## 5. Bistatic Imaging Simulation Based on Configuration Constraints for Aircraft Platform

### 5.1. Simulation Verification of Bistatic SAR Imaging in Different Configurations

In this section, the influence of the spatial distribution of a multi-base SAR platform on imaging resolution is analyzed through the bistatic SAR imaging simulation verification of a cooperative flight trajectory, and then the constraints of the platform configuration are obtained.

Bistatic SAR imaging simulation verification is carried out for different flight paths that meet the configuration requirements. The simulation parameters and index requirements of the system are shown in Table 1.

**Figure 9:**
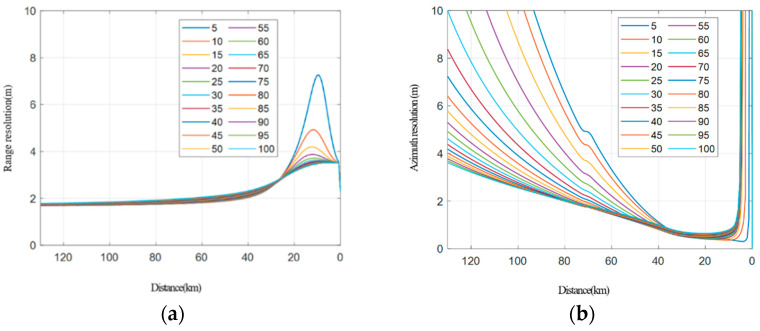
Range resolution and azimuth resolution. (**a**) Range resolution, (**b**) azimuth resolution.

**Figure 10:**
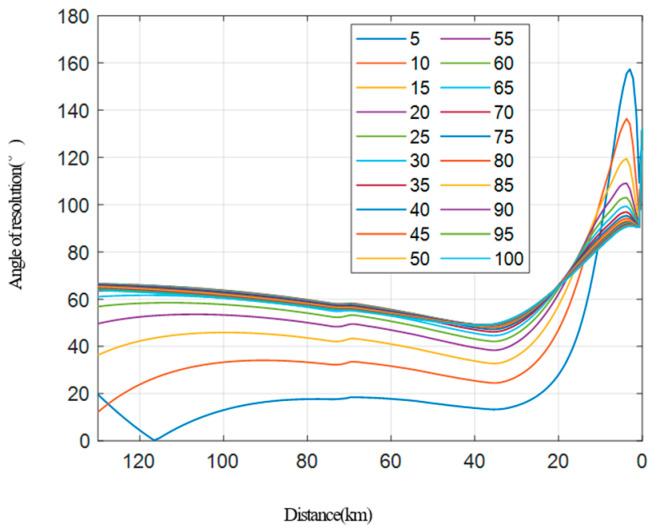
Angle of resolution.

**Figure 11:**
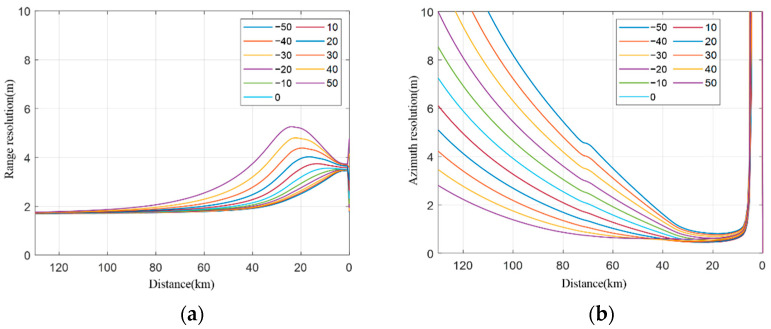
Range resolution and Azimuth resolution. (**a**) Range resolution, (**b**) azimuth resolution.

**Figure 12:**
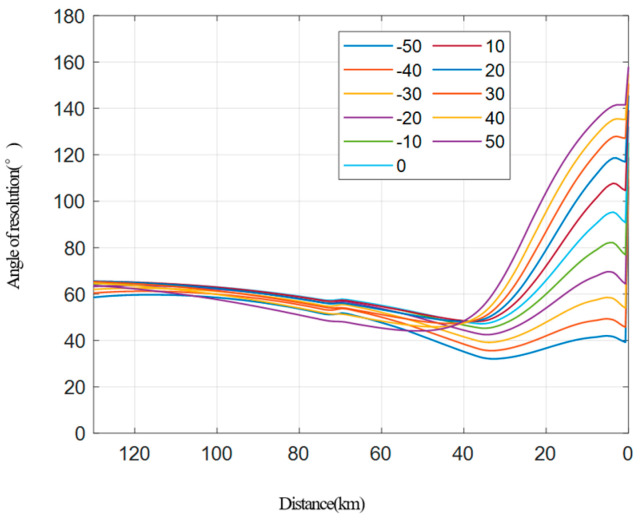
Angle of resolution.

1. The resolution simulation in the case of a fixed longitude distance and variable transverse distance between two platforms.
2. The transverse distance of the two vehicles ranges from 5 km to 100 km, and a simulation is conducted at every 5 km step, as shown in Figure 9. Figure 9a shows how the range resolution varies with the distance between the receiving platform and the target, while Figure 9b shows the performance of the azimuth resolution. Figure 10 shows the relationship between the resolution and the distance between the receiving platform and the target.
3. It can be seen from the simulation that the larger the transverse distance between two vehicles, the better the final range resolution will be, because the larger the interval between two vehicles, the larger the ground projection component provided by the transmitting platform will be. The larger the transverse distance between the two vehicles, the worse the azimuth resolution of the final stage will be, because the transmitting platform with a short interval can provide a larger angular velocity component under the same velocity size. When the transverse distance between two platforms is small, the resolution angle is less than 30° in most cases, while when the transverse distance between the two platforms is large, the resolution angle remains 30° to 150° most of the time (except for the rear period), which meets the requirements of high-resolution imaging.
4. Resolution simulation in the case of a fixed transverse distance and variable longitude distance between two platforms.
5. In the collaborative flight of two platforms, the data transmission distance between the platforms is limited, and the platform spacing is generally within 20–40 km. Here, the transverse distance between the two platforms is fixed at 40 km for the simulation. The position of the receiving platform is fixed, and the distance between the transmitting platform and the receiving platform changes from −50 km to 50 km (negative indicates the backward direction). The simulation analysis is conducted at every 10 km step, and the results are shown in Figure 11. Among them, Figure 11a shows how the range resolution varies with the distance between the receiving platform and the target, while Figure 11b indicates the results of the azimuth resolution. Figure 12 shows the situation that the resolution-included angle in the imaging field varies with the distance between the receiving platform and the target.

As can be seen from the above simulation, the resolution of the whole range becomes better when the transmitting platform is pulled back at a large distance, and the greater the distance is, the higher the resolution is. However, when the transmitting platform is pulled back, the azimuth resolution deteriorates, and the deterioration becomes more obvious at a larger distance. This is mainly because when the transmitting platform is pulled back at a larger distance, the included angle between the ground projection component provided by the transmitting platform and the ground projection component provided by the receiving platform decreases, while the angular velocity component provided by the transmitting platform decreases. In addition, after the transmitting platform is pulled back, the resolution angle of the scene center will decrease correspondingly. The larger the distance is, the smaller the resolution angle of the scene center will be.

On the contrary, when the transmitting platform is pulled forward, the azimuth resolution will improve, while the range resolution will deteriorate (especially at the ending stage). The greater the distance is pulled forward, the azimuth resolution will improve more obviously, while the final range resolution will deteriorate more obviously. This is because, when the transmitting platform moves forward, the azimuth velocity provided by the transmitting platform increases, while the included angle between the ground shadow component provided by the transmitting platform and the ground projection component provided by the receiving platform increases, and it will be even larger than 90° at the end of the stage, which will largely decrease the range resolution.

### 5.2. Configuration Constraints Optimization

By the simulation results that go through all the cooperative flight tracks in this paper, the changing tendencies of the range resolution, azimuth resolution, and resolution angle with transverse and longitude distances between platforms can be obtained.

Regarding the transverse distance, it is recommended to be as large as possible on the basis of ensuring the communication network. When the longitude distance of the platforms keeps constant and the transverse distance changes, it can be seen from the simulation that the larger the transverse distance is, the better the range and the azimuth resolution will be. When the transverse distance between the two platforms is small, the resolution angle is less than 30° most of the time, while when the transverse distance between the two platforms is large, the resolution angle will go between 30° and 150° in the most time (except at the ending period). This will satisfy the imaging requirements.

As to the longitude distance, it is recommended that the transmitting station be separated as large as −10~30 km. When the longitude distance of the platforms changes and the transverse distance is kept constant, the simulation shows that the range and the azimuth resolution have conflicting requirements for the distance and direction of the front and the rear platforms. With a preset resolution constraint, the distance between the platform and the target is analyzed from the flight segment of 80–40 km. For the range resolution, when the platform and the target are 80 km far away, the preset resolution can be met. When the target distance of the platform is 40 km, the distance between the front and the rear transmitting platforms is −50~30 km, which can meet the requirements. For the azimuth resolution, when the platform target distance is 80 km, the longitude distance of the transmitting platforms is −10~50 km, which can meet the resolution requirements; when the platform target distance is 40 km, it can meet the preset resolution requirements. In summary, it is recommended that the two transmitting stations be separated from −10 km to 30 km away.

## 6. Conclusions

Based on the construction of the geometric model of bistatic SAR imaging for an aircraft platform, this paper uses the extended NLCS algorithm to simulate and verify the effect of aircraft bistatic SAR imaging. A geometric model of an aircraft and target is established based on forward-looking bistatic SAR imaging, and a trajectory design method for platform cooperative flight is proposed. Based on the resolution gradient model, the relationship between the precision of the bistatic SAR imaging and the cooperative flight trajectory is studied, and the envelope range of the platform flight position for imaging the preset target point is given under a desirable resolution. The work of this paper will provide a theoretical basis for the implementation of the bistatic SAR imaging algorithm in engineering applications.

## Figures and Tables

**Table 1:** Bistatic SAR forward-looking simulation parameters.

Parameter	Transmitter Down Angle	Receiver Down Angle	Transmitter Squint Angle	Receiver Squint Angle	Transmitter Initial Position	Receiver Initial Position
Value	15.23°	65.49°	−61.88°	89.85°	(1, 0.5, 15) km	(0, 0, 10) km

**Table 2:** Bistatic SAR forward-looking simulation parameters.

Parameter	Central Point (A)		Edge Point (B)		Edge Point (C)	
	Azimuth Direction	Range Direction	Azimuth Direction	Range Direction	Azimuth Direction	Range Direction
PSLR (dB)	−13.20	−13.26	−13.07	−13.31	−13.15	−13.33
ISLR (dB)	−10.07	−9.87	−9.77	−10.21	−9.78	−10.08

## Data Availability

The raw/processed data required to reproduce these findings cannot be shared at this time as the data also forms part of an ongoing study.
